# A Photo-Crosslinked Casein-Tannic Acid System for Enhanced Hair Protection: A Green Chemistry Approach

**DOI:** 10.3390/polym17121585

**Published:** 2025-06-06

**Authors:** Sujin Kyung, Won-Gun Koh, Hyun Jong Lee

**Affiliations:** 1 School of Chemical, Biological and Battery Engineering, Gachon University, 1342 Seongnam-daero, Seongnam-si 13120, Republic of Korea; rudtnwls990802@naver.com; 2 Department of Chemical and Biomolecular Engineering, Yonsei University, 50 Yonsei-ro, Seodaemun-gu, Seoul 03722, Republic of Korea

**Keywords:** casein, tannic acid, photo-crosslinking, hair protection, sustainable cosmetics

## Abstract

Hair is continuously exposed to various damaging factors in daily life, necessitating effective protective strategies that balance efficacy with environmental sustainability. In this study, we developed an environmentally friendly hair protective coating using casein proteins crosslinked with tannic acid via riboflavin phosphate-mediated photo-initiation. Casein solutions containing tannic acid (0.05% *w*/*v*) and riboflavin phosphate (0.01–0.1% *w*/*v*) were prepared and applied to virgin Asian hair, followed by blue light irradiation to initiate crosslinking. The coating formation mechanism was investigated through rheological characterization, which confirmed successful network formation with optimal mechanical stability at a 0.05% tannic acid concentration. Chemical analysis using FTIR spectroscopy revealed subtle but meaningful interactions between the coating components, while SEM analysis demonstrated the coating’s integration with the hair surface. Mechanical property evaluations showed that the photo-crosslinked coating significantly enhanced hair tensile strength by approximately 21% compared to untreated hair, while maintaining appropriate elasticity. Region-specific analysis of stress–strain behavior indicated that the coating extended the initial Hookean region while preserving natural resistance in the post-yield region, creating a balanced enhancement in mechanical properties. This approach offers a promising alternative to conventional hair treatments by utilizing natural, food-grade components and mild processing conditions, addressing growing demands for sustainable hair care solutions that effectively protect against daily damage.

## 1. Introduction

Hair is continuously exposed to various damaging factors in daily life, including physical stress from styling processes, environmental factors such as UV radiation and pollutants, and chemical treatments like bleaching and permanent waving [1,2,3]. These factors compromise the hair’s structural integrity, leading to cuticle damage, decreased mechanical strength, and diminished aesthetic qualities, which collectively affect consumer satisfaction with their hair appearance and feel [4,5].

Current hair protection approaches can be broadly categorized into two groups: temporary surface treatments and permanent chemical modifications. Temporary treatments predominantly rely on silicones (e.g., dimethicone, amodimethicone) and quaternary ammonium compounds that form a hydrophobic layer on the hair surface [6,7]. For instance, studies on silicone emulsions stabilized with various surfactants have shown that while they can improve hair conditioning, most formulations exhibit poor durability, requiring frequent reapplication due to significant removal after just a few wash cycles [6]. Additionally, concerns about the environmental persistence and low biodegradability of these compounds have prompted research into more sustainable alternatives [7].

Permanent chemical modifications offer more durable protection through covalent bonding with hair proteins. Commercial treatments like Brazilian keratin treatments utilize formaldehyde or glyoxylic acid to create crosslinks within the hair structure [8,9]. While effective in providing long-lasting protection, Weathersby and McMichael documented significant concerns regarding formaldehyde exposure, noting that such exposure can lead to adverse health effects, including eye irritation, allergic skin reactions, and respiratory tract irritation [9].

More recently, researchers have explored naturally derived alternatives for hair protection. Chitosan-based coatings have been investigated for cosmetic applications, demonstrating promising biocompatibility, but facing challenges due to limited water solubility, which affects formulation stability and requires chemical modifications to enhance performance [10]. Similarly, other natural-derived materials including phytochemicals have shown potential benefits for strengthening hair fibers, improving cuticle integrity, and reducing breakage [11]. However, these natural ingredients often exhibit inconsistent absorption and limited durability, particularly under wet conditions, requiring formulation adjustments to optimize their effectiveness [11]. These challenges highlight the need for innovative approaches that can enhance the performance of natural materials while maintaining their environmental benefits for hair protection applications.

Our research group has recently developed a successful approach for creating sustainable biopolymer-based films with enhanced durability for color cosmetics applications [12]. This system employed casein and tannic acid (TA) with riboflavin phosphate (RFP)-mediated photo-crosslinking to create stable films that significantly improved the retention of water-soluble dyes on human skin. The films demonstrated excellent biocompatibility, mechanical stability, and resistance to degradation in wet conditions. The success of this biomimetic approach in color cosmetics provides a promising foundation for addressing similar challenges in hair protection, where durability under wet conditions and environmental sustainability are equally critical concerns.

Casein, comprising approximately 80% of milk proteins, represents an ideal candidate for biomimetic hair protection due to its unique structural properties [13]. Its molecular architecture features distinct hydrophobic and hydrophilic domains that enable self-assembly and film formation [14]. The high proline content (approximately 10%) provides conformational flexibility, while the calcium-binding phosphoserine residues offer potential interaction sites for crosslinking agents [15,16]. Notably, casein contains abundant tyrosine residues (approximately 6%) that can participate in crosslinking reactions, particularly under photo-oxidative conditions [12,17]. These characteristics have been exploited in various applications including food packaging, adhesives, and biomedical coatings, with recent extensions into cosmetic formulations [18,19].

Tannic acid (TA), a plant-derived polyphenolic compound abundant in tree bark, offers an environmentally friendly alternative to synthetic crosslinking agents commonly used in commercial formulations [20,21]. With 25 galloyl groups per molecule, TA provides multiple interaction sites for protein binding through both hydrogen bonding and hydrophobic interactions [22,23]. The high density of phenolic hydroxyl groups enables extensive network formation when combined with proteins. Furthermore, TA possesses inherent antioxidant properties that could provide additional protection against oxidative damage to hair [24]. While TA is recognized as a food-grade ingredient with established safety for oral consumption, appropriate handling protocols during formulation are necessary to prevent potential skin, eye, and respiratory irritation, as recommended by regulatory guidelines [25]. The low concentrations typically employed in cosmetic applications (0.01–0.1%) present minimal safety concerns compared to industrial processing levels, positioning TA as a safer alternative to conventional chemical crosslinkers such as formaldehyde-releasing agents.

Previous studies have demonstrated that tannic acid can significantly enhance the mechanical properties of protein structures. Won et al. found that a polyphenol complex containing tannic acid increased the durability and elasticity of damaged hair, indicating effective restoration of the hair cuticle [26]. Additionally, Zhu et al. observed that tannic acid-modified keratin-based hydrogels exhibited enhanced mechanical stability with improved elastic modulus and elongation at break, which they attributed to the formation of extensive hydrogen bonding networks [27]. These findings suggest that TA’s interaction with hair proteins could reinforce the mechanical integrity of hair fibers, potentially providing greater resistance to physical stress and damage.

To address the challenge of achieving stable crosslinking under mild conditions, we employ the riboflavin phosphate (RFP)/blue light system [12,28,29,30,31,32]. Crosslinking systems in current hair care formulations typically rely on aldehydes, carbodiimides, or enzymatic processes, each with significant limitations including toxicity concerns, harsh processing conditions, or prohibitive costs [33,34]. In contrast, RFP-mediated photo-crosslinking operates at ambient temperature, neutral pH, and without generating hazardous byproducts, making it particularly suitable for cosmetic applications where safety and environmental considerations are paramount [31,32]. When exposed to blue light (wavelength 440–480 nm), RFP generates reactive oxygen species that facilitate the formation of dityrosine crosslinks between protein molecules, as demonstrated in our previous work with color cosmetics [12,35]. This controlled crosslinking process is critical for transforming an otherwise plasticizing coating system into a structured reinforcing matrix that can enhance mechanical properties while preserving the natural characteristics of the substrate.

In this study, we present an environmentally friendly approach for hair protection using a casein-based coating system crosslinked by tannic acid and photo-initiated using the RFP/blue light system. Our approach builds upon our successful development of biopolymer-based films for color cosmetics, extending this technology to address the unique challenges of hair protection. The novelty of our approach lies in the synergistic combination of these components: casein provides a biocompatible structural matrix with excellent film-forming properties; tannic acid offers multiple crosslinking sites and inherent antioxidant benefits; and the RFP/blue light system enables controlled, on-demand crosslinking under mild conditions.

We investigate the formation mechanism of these protective coatings through rheological characterization and chemical analysis, examining how the interplay between casein, tannic acid, and photo-initiated crosslinking impacts network formation and mechanical properties. Additionally, we evaluate the coating’s effectiveness in protecting hair from physical damage through region-specific mechanical property analysis, assessing how the coating influences distinct structural regions of the hair fiber’s stress–strain profile. This comprehensive investigation, with particular focus on the initial Hookean region, yield region, and post-yield region of the stress–strain curve, provides deeper insights into the coating’s interaction with different structural elements of hair. The findings establish a foundation for developing next-generation hair care products that align with consumer demands for sustainable formulations while delivering effective protection against daily hair damage.

## 2. Materials and Methods

### 2.1. Materials

Casein from bovine milk, tannic acid (TA), and riboflavin phosphate (RFP) were purchased from Sigma-Aldrich (St. Louis, MO, USA). Phosphate-buffered saline (PBS; pH 7.4) was obtained from Thermo Fisher Scientific (Waltham, MA, USA). Sodium hydroxide (1N-NaOH) was acquired from Deoksan Pure Chemicals (Ansan, Republic of Korea). All chemicals were used as received without further purification.

Natural Asian virgin black hair tresses (length: 25–30 cm, thickness: 60–80 μm) that had never been chemically treated were obtained from Beaulax Co. (BS-B3A, Saitama, Japan). The hair tresses were stored at room temperature under controlled humidity conditions (50 ± 5% relative humidity) and cut to appropriate lengths before experimental use.

### 2.2. Preparation of Casein-Tannic Acid Coating Solutions

The casein solution was prepared by dissolving casein in a mixture of 10% NaOH and deionized water under continuous stirring at 70 °C. The final concentration of casein was set to 10% (*w*/*v*). For the coating solution, tannic acid was added to achieve a final concentration of 0.05% (*w*/*v*), which was determined to be optimal through preliminary rheological analysis. The solution was stirred continuously for 1 h at 70 °C to ensure complete dissolution and homogeneous mixing.

For photo-crosslinking experiments, RFP was added to the prepared solutions immediately before use. The concentration of RFP was set at 0.01% (*w*/*v*) for rheological analysis and 0.1% (*w*/*v*) for hair coating experiments. All solutions were used within 24 h of preparation and stored at 4 °C when not in use. Prior to application, solutions were allowed to reach room temperature.

For safety assessment purposes, the estimated dermal exposure was calculated based on the application protocol: with a typical application volume of 1–2 mL of coating solution containing 0.05% tannic acid, the estimated tannic acid exposure per treatment ranges from 0.5 to 1.0 mg, which is significantly below documented safety thresholds for topical applications. During solution preparation, appropriate personal protective equipment (PPE) including gloves, safety goggles, and adequate ventilation were employed to prevent inhalation of tannic acid particles and minimize skin/eye contact, following standard laboratory safety protocols for polyphenolic compounds.

### 2.3. Rheological Characterization of Coating Solutions

Rheological measurements were performed using a rheometer (MCR 302, Anton Paar, Austria) equipped with a parallel plate geometry (diameter: 25 mm). All measurements were conducted at 37 °C with a gap size of 0.45 mm between the plates. Prior to the frequency sweep measurements, amplitude sweep tests were performed to determine the linear viscoelastic region (LVR). The amplitude sweep was conducted at a constant frequency of 1 Hz with strain ranging from 0.1% to 10%.

Frequency sweep measurements were carried out at a constant strain of 1% (within the LVR) over a frequency range of 0.1–10 Hz. For photo-crosslinking studies, samples containing RFP (0.01%) were first exposed to blue light (458 nm wavelength, 8.47 W power output, 3 cm distance from face-illuminating LED source) for 10 min, and then rheological measurements were performed. Storage modulus (G′) and loss modulus (G″) were recorded as a function of frequency to evaluate the gelation behavior and network formation.

To determine the optimal crosslinker concentration, additional studies were conducted with varying TA concentrations of 0.025%, 0.05%, and 0.1% (*w*/*v*). All measurements were performed in triplicate to ensure reproducibility.

### 2.4. Chemical Analysis of Crosslinked Coating

Fourier transform infrared (FTIR) spectroscopy was performed to analyze the chemical interactions between casein and TA, as well as the effects of the photo-crosslinking process. FTIR spectra were recorded using an ATR-FTIR spectrometer over the wavenumber range of 600–4000 cm^−1^. The background spectrum was collected once before sample measurements.

Three different sample groups were prepared for analysis: casein, casein + TA, and casein + TA + RFP + BL. For the blue light treatment group, samples were exposed to blue light (458 nm, 8.47 W, 3 cm distance) for 10 min prior to freezing. All samples were lyophilized for three days before FTIR analysis. The RFP concentration was maintained at 0.01% for all relevant samples, and TA was used at a 0.05% concentration.

For each measurement, samples were placed directly on the ATR crystal and measured with consistent pressure. Multiple scans were averaged to improve the signal-to-noise ratio, and the resulting spectra were analyzed to identify characteristic peaks associated with crosslinking interactions and structural changes in the protein network.

### 2.5. Hair Coating and Treatment Process

Hair coating was performed using the prepared casein + TA solution with RFP. The coating process began with the addition of RFP (0.1%) to the casein + TA solution immediately before use. Natural virgin hair tresses were immersed in this solution for 60 min at room temperature to ensure uniform coating. After the immersion period, individual hair strands were carefully separated and exposed to blue light (458 nm, 8.47 W, 3 cm distance) for 10 min to initiate the photo-crosslinking process.

Following the photo-crosslinking step, the coated hair samples were gently washed with PBS for 5 min to remove any unbound materials. The washed samples were then dried in an incubator at 37 °C for 24 h. For control experiments, hair samples were also prepared using coating solutions without RFP and without blue light exposure, following the same coating and washing procedures. All coating processes were conducted under controlled laboratory conditions (temperature: 25 ± 2 °C; relative humidity: 50 ± 5%) to ensure reproducibility. The coated hair samples were stored in a controlled environment until further characterization.

Based on the coating protocol and hair sample dimensions, the final tannic acid loading on treated hair was estimated to be approximately 0.1–0.2 mg per gram of hair, representing minimal systemic exposure risk during normal use.

### 2.6. Surface Morphology Analysis

The surface morphology of coated and uncoated hair samples was examined using scanning electron microscopy (SEM, SU8600, Hitachi, Tokyo, Japan) at the Smart Materials Research Center for IoT at Gachon University. Hair samples were prepared by cutting 12 cm lengths of treated and untreated hair strands. The coating process followed the procedure described in Section 2.5. After the coating and drying processes, samples were mounted on aluminum stubs using conductive carbon tape.

Prior to SEM analysis, all samples were sputter-coated with a thin layer of platinum under vacuum for 60 s to enhance conductivity. SEM observations were conducted at an accelerating voltage of 5 kV under high-vacuum conditions. Representative images were selected for comparative analysis of surface features between different treatment groups.

### 2.7. Mechanical Properties Analysis

The mechanical properties of the hair samples were evaluated using a universal testing machine (UTM, Instron, Norwood, MA, USA). Hair samples were prepared by cutting strands to 12 cm lengths and treating them following the coating process described in Section 2.5. After treatment, the samples were washed with PBS for 10 min and dried in an oven at 37 °C for 24 h. The tested samples included untreated hair, casein + TA-coated hair, and casein + TA + RFP + BL-coated hair.

For tensile testing, the effective length between grips was set to 5 cm. The 12 cm sample length allowed for the exclusion of root and tip regions, which typically exhibit non-uniform properties, while the 5 cm grip distance ensured testing in the uniform mid-shaft region. The ends of each hair strand were carefully wrapped with paper to prevent slippage during testing. Tests were performed at a constant extension rate of 30 mm/min, established from our previous hair testing protocols to provide optimal measurement sensitivity while avoiding rate-dependent artifacts [31,32]. Testing was conducted at room temperature under controlled humidity conditions (50 ± 5% relative humidity). Stress–strain curves were recorded, and parameters including tensile strength, Young’s modulus, and elongation at break were calculated.

To gain deeper insights into the protective effects of the coating on specific structural elements of hair, we conducted region-specific mechanical analysis of the stress–strain curves. Three distinct regions were identified and separately analyzed based on established hair mechanics research: (1) the initial Hookean region, representing the elastic response of the α-keratin helices; (2) the yield region, corresponding to the α-to-β transformation of keratin; and (3) the post-yield region, representing the extension of the transformed β-keratin structure [36].

For each region, the regional elastic modulus was calculated as the slope of the stress–strain curve within the defined strain range. This analysis allowed for a more detailed understanding of how the coating treatment affects different structural components of the hair fiber during mechanical deformation. All mechanical tests were performed with at least five samples per group to ensure statistical reliability.

### 2.8. Statistical Analysis

Statistical analysis was conducted to determine the significance of the results obtained from the various tests. All experiments were performed in triplicate, except for mechanical property measurements which were performed with five replicates (*n* = 5). Data were expressed as the mean ± standard deviation. Comparisons between groups were made using one-way analysis of variance (ANOVA) followed by Tukey’s post hoc test where appropriate, using GraphPad Prism 10 software. A *p*-value of less than 0.05 was considered statistically significant. Statistical significance is denoted as follows: * *p* < 0.05 and ** *p* < 0.01.

## 3. Results and Discussion

### 3.1. Preparation and Characterization of Photo-Crosslinked Casein-TA Coating System

**Figure 1:**
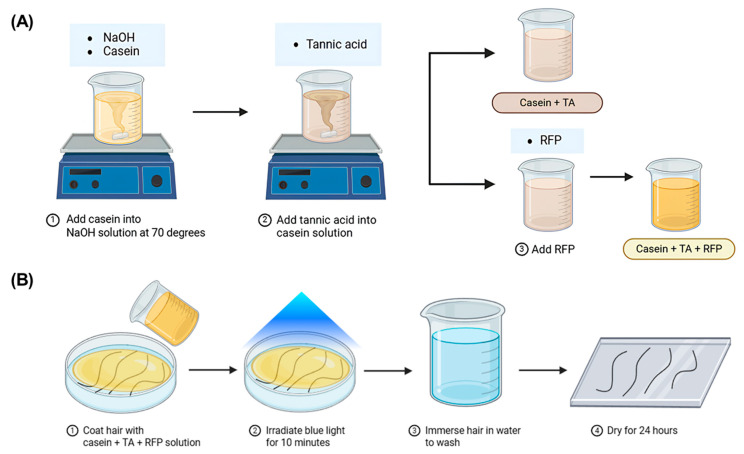
A schematic illustration depicting the hair protection strategy. (**A**) The preparation process of the Casein-Tannic acid–riboflavin phosphate (Casein + TA + RFP) coating solution. (**B**) The hair coating procedure.

This study presents the development of a Casein-Tannic acid photo-crosslinked coating system and evaluates its protective effects on hair. Figure 1 systematically illustrates the preparation process and application methodology of this coating system. Figure 1A depicts the stepwise preparation protocol: Initially, casein is solubilized in NaOH solution at 70 °C to ensure complete dissolution and denaturation of the protein structure, thereby facilitating subsequent interactions with crosslinking agents. Subsequently, tannic acid (TA) is incorporated into the casein solution under continuous agitation, promoting the formation of initial non-covalent interactions between casein’s peptide chains and TA’s multiple phenolic hydroxyl groups. Finally, riboflavin phosphate (RFP) is added as a photoinitiator, completing the formulation of the coating solution.

Figure 1B demonstrates the practical application protocol developed for hair treatment: The prepared casein + TA + RFP solution is first applied to hair samples through immersion, ensuring uniform coating of individual hair strands. After coating, the samples are exposed to blue light irradiation for 10 min. This critical photo-activation step triggers the RFP-mediated crosslinking reaction between casein and TA, transforming the initially reversible physical interactions into a stable, covalently crosslinked network structure on the hair surface. Following the crosslinking process, the samples are immersed in water to wash away and remove any unbound material. The treated hair is then dried under controlled conditions (37 °C for 24 h) to stabilize the coating structure.

This optimized protocol represents a biomimetic approach to hair protection, utilizing natural proteins and polyphenolic compounds to create a functional protective layer that maintains strong adhesion to the hair surface while providing enhanced resistance to environmental stressors, particularly under challenging wet conditions where hair is most vulnerable to mechanical damage.

**Figure 2:**
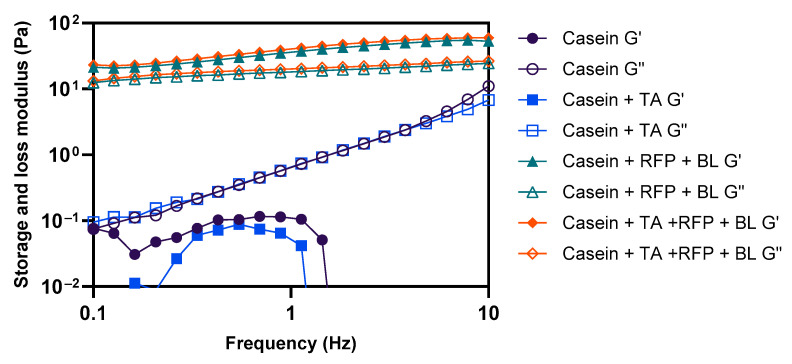
Rheological characterization of casein-based formulations. Frequency sweep analysis showing storage modulus (G′) and loss modulus (G″) for different formulations: casein alone, casein with tannic acid (TA, 0.05%), casein with riboflavin phosphate and blue light irradiation (casein + RFP + BL), and complete system (casein + TA + RFP + BL), demonstrating transition from solution to gel state upon photo-initiated crosslinking.

The rheological properties of the casein-based formulations were comprehensively analyzed to elucidate the network formation mechanism and confirm successful photo-crosslinking. As illustrated in Figure 2, the frequency-dependent viscoelastic behavior of various casein formulations revealed distinctive characteristics that correlate with their structural organization at the molecular level.

For casein alone and casein-TA formulations, the loss modulus (G″) consistently exceeded the storage modulus (G′) across the examined frequency spectrum, indicating a predominant liquid-like behavior characteristic of uncrosslinked polymer solutions. The storage modulus values for these formulations were extremely low, falling below the measurable range, which further confirms the absence of a stable network structure.

In contrast, the incorporation of riboflavin phosphate followed by blue light irradiation (casein + RFP + BL) induced a marked enhancement in both moduli values, with G’ values increasing by approximately two orders of magnitude compared to the non-irradiated samples. Most significantly, the storage modulus surpassed the loss modulus throughout the frequency range examined, exhibiting the characteristic rheological signature of a viscoelastic gel network. This inversion of the G′/G″ relationship provides definitive evidence of successful photo-initiated crosslinking, wherein the blue light activation of RFP generates reactive species that facilitate intermolecular bond formation between casein molecules.

The complete system, incorporating both TA (0.05%) and RFP under blue light irradiation (casein + TA + RFP + BL), demonstrated the most pronounced gel-like behavior, with substantially elevated G’ values that maintained frequency independence—a hallmark of robust crosslinked networks. The superior mechanical properties of this formulation can be attributed to the synergistic effect of TA, which provides additional crosslinking sites through its multiple phenolic hydroxyl groups, thereby enhancing the density and strength of the network structure formed during the photo-crosslinking process.

These rheological findings conclusively demonstrate the successful transformation from a liquid solution to a solid-like gel network through the photo-initiated crosslinking mechanism, confirming the viability of our approach for creating stable protective coatings on hair surfaces.

**Figure 3:**
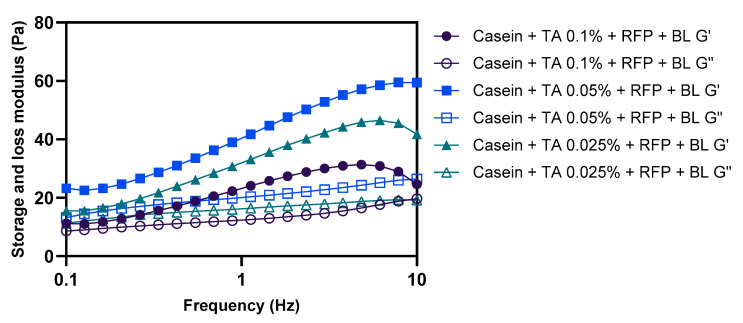
Effect of TA concentration on rheological properties of photo-crosslinked casein networks. Frequency sweep measurements comparing storage modulus (G′) and loss modulus (G″) of casein formulations with different TA concentrations (0.025%, 0.05%, and 0.1% *w*/*v*) after blue light irradiation with RFP.

The concentration of crosslinking agent represents a critical parameter in determining the mechanical properties and structural integrity of protein-based networks. To optimize the formulation, we investigated the effect of varying TA concentrations (0.025%, 0.05%, and 0.1% *w*/*v*) on the rheological properties of photo-crosslinked casein networks. As illustrated in Figure 3, G′ consistently exceeded G″ across all tested formulations, confirming the formation of gel networks regardless of TA concentration. However, the magnitude of the moduli values and their frequency dependence exhibited significant variations across different TA concentrations. The formulation containing 0.05% TA demonstrated markedly superior rheological properties compared to those observed with 0.025% TA and 0.1% TA concentrations.

Careful examination of the frequency dependence profiles reveals important insights into the network structure at different TA concentrations. At 0.025% TA, the relatively lower G’ values and moderate frequency dependence indicate insufficient crosslinking density for optimal network formation. This can be attributed to the limited availability of phenolic hydroxyl groups for interaction with casein molecules, resulting in a less rigid structure with fewer effective crosslinking points.

Interestingly, at a 0.1% TA concentration, despite showing relatively smaller variations in G′ across the frequency range, the absolute G′ values were significantly lower than at 0.05% and 0.025% TA. This seemingly contradictory behavior can be explained by two primary mechanisms: First, excess TA likely forms self-aggregates through π-π stacking and hydrogen bonding between adjacent phenolic rings, effectively reducing the concentration of TA available for productive crosslinking with casein [37,38,39]. Second, high concentrations of TA can reduce the mobility and flexibility of casein molecules through extensive non-covalent interactions (hydrogen bonding and hydrophobic interactions) prior to photo-crosslinking, restricting their ability to adopt optimal conformations for efficient crosslinking during blue light irradiation [40,41,42]. This premature restriction of protein mobility would result in a mechanically weaker network, consistent with the observed rheological profile.

The formulation with 0.05% TA exhibited the highest absolute G′ values, representing an optimal balance between crosslinking density and molecular mobility. At this concentration, sufficient TA is available for effective crosslinking without significantly compromising the conformational flexibility of casein molecules during the photo-crosslinking process. This allows for the formation of a network with both high mechanical strength and appropriate viscoelastic properties for coating applications.

These findings are consistent with previous studies on protein–polyphenol interactions, which have demonstrated concentration-dependent effects where excessive polyphenol concentrations can lead to protein aggregation and reduced functionality due to extensive surface binding and conformational restrictions [43,44].

Based on these comprehensive rheological analyses, the formulation containing 10% casein, 0.05% TA, and 0.01% RFP was identified as optimal for hair coating applications. This composition provides the ideal balance between sufficient crosslinking density for mechanical stability and appropriate molecular mobility for effective network formation during photo-crosslinking. All subsequent experiments in this study were conducted using this optimized formulation to ensure consistent and reliable performance of the protective coating system.

### 3.2. Chemical Characterization of Crosslinked Casein

**Figure 4:**
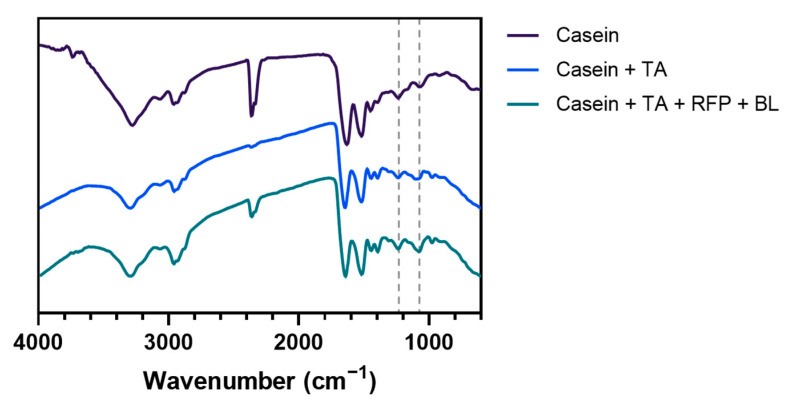
FT-IR spectra of casein, casein + TA, and casein + TA + RFP + BL formulations, showing the characteristic absorption bands across the wavenumber range of 4000–600 cm^−1^. The spectra demonstrate subtle changes in peak intensities and positions, particularly in the amide and hydroxyl regions, indicating molecular interactions between components.

The molecular interactions and potential chemical modifications in the casein-TA photo-crosslinked system were investigated using Fourier transform infrared (FT-IR) spectroscopy. Figure 4 presents the comparative spectra of casein alone, casein + TA, and casein + TA + RFP + BL across the wavenumber range of 4000–600 cm^−1^.

All three formulations exhibited the characteristic protein absorption bands associated with casein structure [12,45]. The broad peak observed in the 3300–3500 cm^−1^ region corresponds to O-H and N-H stretching vibrations, representing hydroxyl groups and peptide bonds, respectively. The prominent bands in the mid-infrared region include amide I (≈1650 cm^−1^, C=O stretching), amide II (≈1550 cm^−1^, N-H bending and C-N stretching), and the fingerprint region below 1500 cm^−1^ containing complex overlapping bands from various functional groups.

More detailed examination of the FTIR spectra reveals subtle but consistent changes between treatment groups. The hydroxyl region (3300–3500 cm^−1^) shows slight peak broadening in treated samples compared to pure casein, indicating enhanced hydrogen bonding interactions between TA’s phenolic hydroxyl groups and casein’s peptide bonds. The casein + TA spectrum displays minor changes in the intensity and broadening of this region, suggesting increased hydrogen bonding interactions. This broadening trend continues in the photo-crosslinked sample, supporting the formation of crosslinked networks.

Additionally, minor shifts in the amide I and II regions indicate potential conformational adjustments in the protein structure upon TA binding [45]. The photo-crosslinked sample (casein + TA + RFP + BL) exhibits further subtle modifications in peak profiles, particularly in the fingerprint region (1000–1500 cm^−1^) where several small peaks show intensity variations compared to non-irradiated controls. These minor variations in the fingerprint region further support the formation of intermolecular associations, though these changes remain modest due to the low TA concentration relative to the bulk casein matrix.

While these spectral changes are not dramatic, they are consistent with limited covalent crosslinking between oxidized phenolic groups of TA and nucleophilic amino acid residues in casein. The relatively modest spectral differences observed among the three formulations can be attributed to several factors: (1) the high concentration of casein (10%) relative to TA (0.05%), resulting in crosslinking that affects only a small fraction of the total protein content; (2) the predominance of non-covalent interactions in the system, which produce more subtle spectral changes than extensive covalent modifications; and (3) the localized nature of the crosslinking reactions, which may not significantly alter the overall secondary structure of casein.

While these spectroscopic changes are subtle, they provide evidence of molecular-level interactions that contribute to the network formation observed in rheological studies. The modest nature of FTIR changes, despite measurable functional improvements, reflects the surface-localized nature of our crosslinking approach and the inherent limitations of bulk characterization techniques for thin coating systems. These spectroscopic observations complement our rheological findings, where significant enhancements in mechanical properties were demonstrated. The FTIR results suggest that while chemical modifications are subtle at the molecular level, these limited but targeted crosslinks between casein and TA molecules create an effective three-dimensional network structure. This illustrates how precisely positioned intermolecular connections can substantially influence physical properties without requiring extensive chemical alteration of the protein backbone. The photo-crosslinking mechanism thus efficiently translates minimal chemical changes into notable mechanical reinforcement, optimizing the performance of the casein + TA protective coating system.

### 3.3. Surface Morphology Analysis of Coated Hair

**Figure 5:**
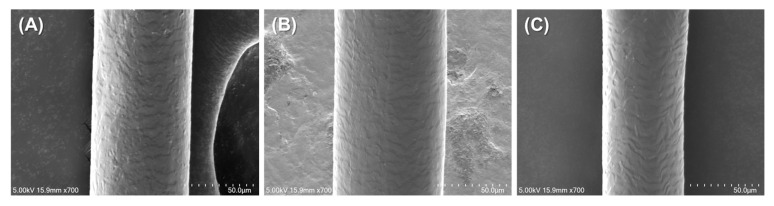
SEM images of hair surface morphology: (**A**) untreated hair, (**B**) casein + TA-treated hair, and (**C**) casein + TA + RFP + BL-treated hair. Scale bars represent 50 μm. Images were obtained at 700× magnification with an accelerating voltage of 5.00 kV.

The surface morphology of treated and untreated hair samples was examined using scanning electron microscopy (SEM) to evaluate the effect of coating treatments on hair surface characteristics. Figure 5 presents representative SEM images of untreated hair, casein + TA-treated hair, and photo-crosslinked casein + TA + RFP + BL-treated hair.

The SEM analysis revealed subtle but meaningful differences between the treatment groups. The untreated hair (Figure 5A) exhibits the typical natural cuticle structure with clearly defined scale patterns and a characteristic textured surface. This observation confirms that the virgin hair samples used in this study were in good condition prior to treatment, providing a suitable baseline for evaluating coating effects.

The casein + TA-treated hair (Figure 5B) shows preservation of the underlying cuticle structure while displaying evidence of surface modification. The natural scale patterns remains visible, but with additional material deposition that appears to partially fill the gaps between cuticle scales, resulting in a slightly modified surface texture compared to the untreated control.

The photo-crosslinked casein + TA + RFP + BL-treated hair (Figure 5C) demonstrates the most notable surface modification while still maintaining the fundamental cuticle architecture. The surface appears smoother than both the untreated and non-crosslinked samples, suggesting more uniform coating distribution and better integration with the hair surface through the photo-crosslinking process.

Importantly, the preservation of cuticle scale visibility across all treated samples, combined with only subtle surface modifications, indicates that the coating forms a very thin layer. Based on these SEM observations, we estimate the coating thickness to be in the sub-micrometer range, likely between 100 and 500 nm. This thin coating layer explains several key observations: (1) the natural hair surface contour and cuticle structure are preserved, (2) no distinct coating layer is visible as a separate phase, and (3) the coating appears to integrate with rather than mask the hair’s natural surface features.

A significantly thicker coating (micrometer scale) would have resulted in complete masking of the cuticle features and dramatically altered surface morphology, creating a smooth, uniform appearance that was not observed in our analysis. Instead, the subtle surface modifications observed suggest that the coating system works through molecular-level interactions that enhance hair properties without dramatically altering its natural structure.

These morphological findings support our mechanical testing results, where significant functional improvements were achieved despite minimal visible coating thickness. The thin, well-integrated coating layer demonstrates that effective hair protection can be accomplished through precise molecular-level modifications rather than thick physical barriers, aligning with our approach of using natural, food-grade components in optimized concentrations.

### 3.4. Mechanical Properties of Coated Hair Under Dry Conditions

**Figure 6:**
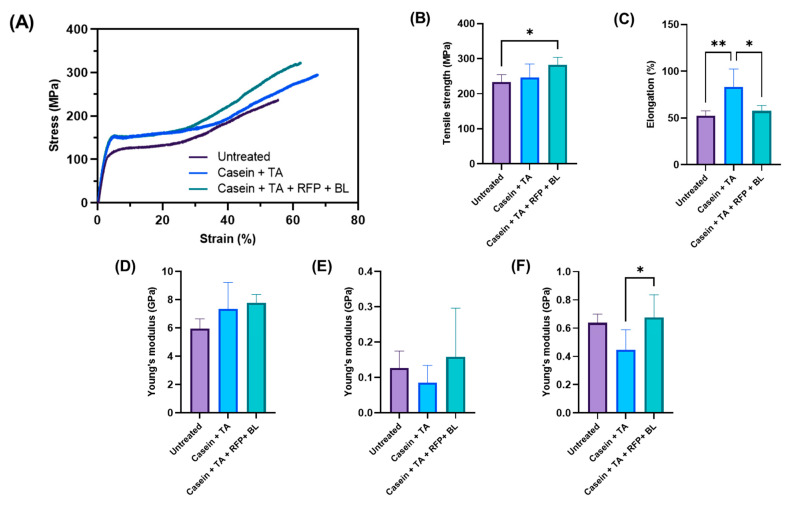
Mechanical properties of untreated and coated hair under dry conditions. (**A**) Representative stress–strain curves showing three distinct mechanical response regions (Region 1: initial Hookean region, 0–2% strain; Region 2: yield region, 10–20% strain; Region 3: post-yield region, 40–50% strain), (**B**) tensile strength, (**C**) elongation at break, (**D**) Young’s modulus in Region 1 (initial Hookean region), (**E**) Young’s modulus in Region 2 (yield region), and (**F**) Young’s modulus in Region 3 (post-yield region) for untreated hair, casein + TA-coated hair, and casein + TA + RFP + BL-coated hair. Error bars represent standard deviation (*n* = 5). Statistical significance: * *p* < 0.05 and ** *p* < 0.01.

The mechanical properties of hair samples were comprehensively evaluated to assess the effect of coating treatments on hair strength and elasticity under dry conditions. Figure 6 presents the mechanical testing results, including region-specific analyses of the stress–strain behavior that provide deeper insights into the structural modifications imparted by the coating treatments.

The stress–strain curves (Figure 6A) reveal three distinct mechanical response regions across all sample groups, each representing different structural phenomena within the hair fiber: the initial Hookean region (Region 1, 0–2% strain) corresponding to the elastic stretching of α-keratin helices; the yield region (Region 2, 10–20% strain) representing the α-to-β transformation of keratin; and the post-yield region (Region 3, 40–50% strain) reflecting the extension of transformed β-keratin structures before ultimate failure [36].

Tensile strength (Figure 6B) shows improvement in the coated samples, with values increasing from untreated hair (233 ± 19 MPa) to casein + TA-coated hair (247 ± 34 MPa), and reaching the highest level in the photo-crosslinked casein + TA + RFP + BL-treated hair (283 ± 19 MPa, *p* < 0.05 compared to untreated). This statistically significant enhancement (approximately 21% increase) in the photo-crosslinked samples indicates that the treatment effectively reinforces the hair structure. The tensile strength improvement correlates with the extension of the initial Hookean region observed in the stress–strain curves, suggesting that the coating allows the hair to maintain its elastic behavior over a wider strain range before initiating structural transitions.

Elongation at break (Figure 6C) exhibits significant variation among treatment groups. Untreated hair shows an elongation of 53 ± 5%, while casein + TA-treated hair demonstrates a substantially increased elongation of 83 ± 17% (*p* < 0.01 compared to untreated). In contrast, the photo-crosslinked casein + TA + RFP + BL-treated hair exhibits an elongation of 58 ± 5%, which is significantly lower than that of the non-crosslinked casein + TA treatment (*p* < 0.05) but comparable to that of untreated hair. This pattern suggests a fundamental difference in how the two coating systems interact with the hair structure: the non-crosslinked coating introduces a plasticizing effect that enhances deformability, while the photo-crosslinked coating provides reinforcement without compromising the hair’s native extensibility characteristics.

Region-specific analysis of the stress–strain curves revealed particularly illuminating insights into the coating’s effects on hair mechanics. In the initial Hookean region (Figure 6D), although not reaching statistical significance, Young’s modulus values show an increasing trend from untreated hair (5.97 ± 0.61 GPa) to casein + TA-coated hair (7.35 ± 1.67 GPa) and photo-crosslinked casein + TA + RFP + BL-treated hair (7.76 ± 0.54 GPa). More importantly, both coated sample groups exhibit an extended initial Hookean region compared to the untreated control. This extension of the elastic region represents a critical enhancement, as it allows the hair to withstand higher stresses while maintaining reversible deformation behavior, thereby establishing a foundation for the observed increase in tensile strength.

The extended initial Hookean region can be attributed to the tannic acid component in both coating systems. Tannic acid, with its multiple phenolic hydroxyl groups, likely forms extensive hydrogen bonds with the surface proteins of the hair cuticle, creating a supportive network that delays the onset of plastic deformation [24,46]. This effect is consistent with previous studies on polyphenol–protein interactions, which have demonstrated the ability of polyphenols to stabilize protein structures through multiple non-covalent interactions [47].

In the yield region (Figure 6E), Young’s modulus values show slight variations: untreated hair (0.13 ± 0.04 GPa), casein + TA-coated hair (0.09 ± 0.04 GPa), and photo-crosslinked casein + TA + RFP + BL-treated hair (0.16 ± 0.12 GPa). While these differences did not reach statistical significance due to the inherent variability in this transitional region, the trend suggests that the non-crosslinked casein + TA coating reduces resistance during the α-to-β transformation, facilitating the structural transition and contributing to the increased elongation observed in Figure 6C. Conversely, the photo-crosslinked coating appears to maintain or slightly increase the resistance to structural transformation compared to untreated hair.

The differences are also observed in the post-yield region (Figure 6F), where Young’s modulus values differ significantly between treatment groups: untreated hair (0.64 ± 0.06 GPa), casein + TA-coated hair (0.45 ± 0.13 GPa), and photo-crosslinked casein + TA + RFP + BL-treated hair (0.67 ± 0.14 GPa, *p* < 0.05 compared to casein + TA). The significantly lower post-yield modulus in the casein + TA group aligns with its increased elongation behavior, confirming the plasticizing effect of the non-crosslinked coating on the extended β-keratin structure. In contrast, the photo-crosslinked coating maintains a post-yield modulus comparable to that of untreated hair, indicating that the RFP-mediated crosslinking effectively counteracts the plasticizing effect of the casein + TA system while still providing strengthening benefits.

The distinctive mechanical profiles observed across the three regions of the stress–strain curve reveal the complex mechanisms through which the coating treatments modify hair properties. The non-crosslinked casein + TA coating appears to function primarily as a plasticizing agent that increases deformability by reducing resistance in both the yield and post-yield regions. While this may be advantageous for improving hair flexibility, the reduced structural resistance could compromise dimensional stability and resilience, particularly under challenging environmental conditions.

In contrast, the photo-crosslinked casein + TA + RFP + BL coating achieves a remarkable balance: it enhances tensile strength through the extension of the initial Hookean region while maintaining appropriate resistance in the post-yield region, thus preserving the natural mechanical integrity of the hair fiber. This balanced enhancement can be attributed to the formation of a structured network through RFP-mediated crosslinking, which transforms the otherwise plasticizing casein + TA system into a reinforcing matrix that complements the hair’s intrinsic mechanical properties.

At the molecular level, several mechanisms likely contribute to these observed effects. The riboflavin phosphate, when activated by blue light, generates reactive oxygen species that facilitate the formation of dityrosine crosslinks between casein molecules and potentially between casein and surface proteins of the hair cuticle [35,48]. These covalent crosslinks create a mechanically robust network that maintains its structural integrity even under high-strain conditions. Additionally, the photo-crosslinked coating may form a more coherent interface with the hair surface, effectively distributing applied stresses and preventing localized failure initiation.

When considered alongside the chemical and morphological analyses, these mechanical findings provide a comprehensive understanding of our coating system’s performance. The FTIR results suggest subtle but meaningful interactions between coating components and hair, which, while not dramatically altering bulk chemistry, are sufficient to create effective bonding with the hair surface. The SEM analysis, which showed minimal visible thickness of the coating, indicates that the system functions not as a distinct external layer but as an integrated structural element that works synergistically with the hair’s native architecture.

These findings collectively demonstrate that our environmentally friendly, photo-crosslinked casein + TA + RFP + BL coating system provides significant improvements in hair mechanical properties through region-specific modifications of the stress–strain behavior. The coating enhances tensile strength without compromising natural elasticity, extends the reversible deformation range, and maintains appropriate resistance in the post-yield region. This balanced approach to mechanical modification represents a significant advantage over conventional treatments that often sacrifice certain mechanical properties to enhance others. Furthermore, the critical role of photo-initiated crosslinking in achieving optimal mechanical properties highlights the innovative nature of this approach. By utilizing riboflavin phosphate—a natural, food-grade photo-initiator—and blue light to trigger controlled crosslinking, we have developed a system that transforms a basic casein + TA coating into a high-performance protective treatment that enhances hair strength while preserving its essential mechanical characteristics.

From a safety standpoint, our coating system exclusively employs food-grade ingredients (casein, tannic acid, riboflavin phosphate) that present minimal risk to human health, in contrast to conventional treatments containing potentially harmful chemicals such as formaldehyde-releasing agents. The mild photo-crosslinking conditions eliminate exposure to harsh processing environments, enhancing safety for both manufacturers and end-users. This environmentally friendly approach aligns with the growing demand for sustainable and safe hair care solutions while delivering measurable performance improvements.

## 4. Conclusions

In this study, we developed an environmentally friendly approach for hair protection using a casein-based coating system crosslinked by tannic acid and photo-initiated using the riboflavin phosphate/blue light system. The rheological characterization demonstrated successful formation of a stable network through photo-crosslinking, with optimal mechanical properties achieved at a 0.05% tannic acid concentration. Chemical analysis through FTIR spectroscopy, while showing subtle changes, confirmed the interactions between casein and tannic acid, supporting the network formation observed in the rheological studies. Mechanical property evaluations revealed that the photo-crosslinked casein-TA-RFP coating significantly enhanced hair tensile strength by approximately 21% compared to untreated hair while maintaining the hair’s natural extensibility. Region-specific analysis of the stress–strain behavior provided deeper insights into the coating’s mechanism of action, demonstrating that it extended the initial Hookean region while preserving appropriate resistance in the post-yield region. This balanced enhancement in mechanical properties distinguishes our approach from conventional treatments that often sacrifice certain mechanical characteristics to improve others. The environmentally friendly nature of our coating system, utilizing food-grade, biodegradable components and mild processing conditions, addresses the growing consumer demand for sustainable hair care solutions. This work establishes a foundation for next-generation hair protection strategies that effectively balance performance, safety, and environmental considerations, with potential applications extending beyond hair care to other protein-based fiber protection needs.

## Data Availability

The original contributions presented in this study are included in the article.
